# Thyroid dysfunction and sarcopenia: a two-sample Mendelian randomization study

**DOI:** 10.3389/fendo.2024.1378757

**Published:** 2024-09-05

**Authors:** Jiaxin Wei, Shuanglong Hou, Peng Hei, Gang Wang

**Affiliations:** ^1^ Department of Sport Rehabilitation, School of Graduate, Xi'an Physical Education University, Xi’an, Shaanxi, China; ^2^ School of Sports and Health Science, Xi’an Physical Education University, Xi’an, Shaanxi, China

**Keywords:** Mendelian randomization, sarcopenia, hyperthyroidism, hypothyroidism, low hand grip strength, appendicular lean mass, walking pace

## Abstract

**Objective:**

Observational studies have shown positive associations between thyroid dysfunction and risk of sarcopenia. However, the causality of this association remains unknown. This study aimed to evaluate the potential causal relationship between thyroid dysfunction and sarcopenia using Mendelian randomization (MR).

**Methods:**

This study collected pooled data from genome-wide association studies focusing on thyroid dysfunction and three sarcopenia-related features: low hand grip strength, appendicular lean mass (ALM), and walking pace, all in individuals of European ancestry. The primary analytical method used was inverse-variance weighted, with weighted median and MR-Egger serving as complementary methods to assess causal effects. Heterogeneity and pleiotropy tests were also performed, and the stability of the results was evaluated using the Leave-one-out.

**Results:**

The MR analysis indicated that hyperthyroidism could lead to a significant decrease in ALM in the extremities (OR = 1.03; 95% CI = 1.02 to 1.05; *P* < 0.001). The analysis also found that hypothyroidism could cause a notable reduction in grip strength (OR = 2.03; 95% CI = 1.37 to 3.01; *P* < 0.001) and walking pace (OR = 0.83; 95% CI = 0.77 to 0.90; *P* < 0.001). There was a significant association between subclinical hyperthyroidism and a reduced walking pace (OR = 1.00; 95% CI = 0.99 to 1.00; *P* = 0.041).

**Conclusion:**

This study provides evidence that hyperthyroidism, hypothyroidism, and subclinical hyperthyroidism can all increase the risk of sarcopenia.

## 1 Introduction

Sarcopenia is a progressive and systemic disease that affects skeletal muscles, leading to a heightened risk of adverse outcomes such as falls, fractures, reduced physical function, and increased mortality (1). Epidemiological studies demonstrated that sarcopenia affects between 10%-27% of the aged population worldwide (2). This condition poses a significant health challenge on a global scale, leading to substantial medical and economic burdens on society (3). However, most patients with sarcopenia do not receive adequate medical or health intervention. Depending on the cause, sarcopenia is divided into two types. Primary sarcopenia is associated with aging and involves the biological basis of aging. Secondary sarcopenia can be traced back to specific causes such as malnutrition, physical inactivity, and chronic disease (4). Regardless of the phenotype, the main pathological contributors are inflammation, oxidative stress, protein anabolic imbalance, and hormonal disorders (5).

Thyroid hormones are crucial for maintaining metabolism and are involved in the regulation of growth, development, and substance metabolism. The skeletal muscle is its main target organ, and thyroid hormone signaling is involved in skeletal muscle development, plasticity and repair by regulating the expression of core genes related to skeletal muscle homeostasis, function and metabolism (6). A previous study showed that declining thyroid hormone levels associated with aging can lead to decreased type II muscle fibers (7). In patients with subclinical thyroid disease, serum thyroid-stimulating hormone levels are associated with sarcopenia and its components in a U-shaped relationship (8). Loss of muscle mass and decreased muscle function are common in patients with hyperthyroidism. One study found that patients receiving effective hyperthyroidism treatment experienced significant recovery in skeletal muscle mass index and grip strength (9). A possible explanation is that abnormal thyroid hormone levels lead to abnormal mitochondrial activity, oxidative phosphorylation, and oxygen consumption, which affect the resting metabolic rate of skeletal muscle. This in turn affects the catabolism of protein metabolism, causing muscle atrophy and decreased muscle function (10). Additionally, exposure of satellite cells to abnormal thyroid hormone levels leads to a decrease in Pax7 cells, which is detrimental to muscle regeneration (11). These studies point in the same direction, highlighting the association of thyroid disease with sarcopenia. However, most of these studies were cross-sectional, had small sample sizes, and were limited by confounding factors and reverse causality. Determining whether there is a causal relationship between these two is critical for the identification of secondary sarcopenia subtypes and their targeted therapeutic strategies.

Mendelian randomization (MR) is a research method that leverages genetic data, using single-nucleotide polymorphisms (SNPs) as instrumental variables (IVs). This approach effectively mitigates the effects of confounders and reverse causation, providing more robust causal inferences (12, 13). To date, no MR studies have been published exploring the causal relationship between thyroid dysfunction and sarcopenia. Therefore, this study aims to fill this gap by analyzing the potential causal relationship between thyroid dysfunction and sarcopenia using MR methods to provide a theoretical basis for prevention and targeted intervention in sarcopenia.

## 2 Materials and methods

### 2.1 Study design

**Figure 1:**
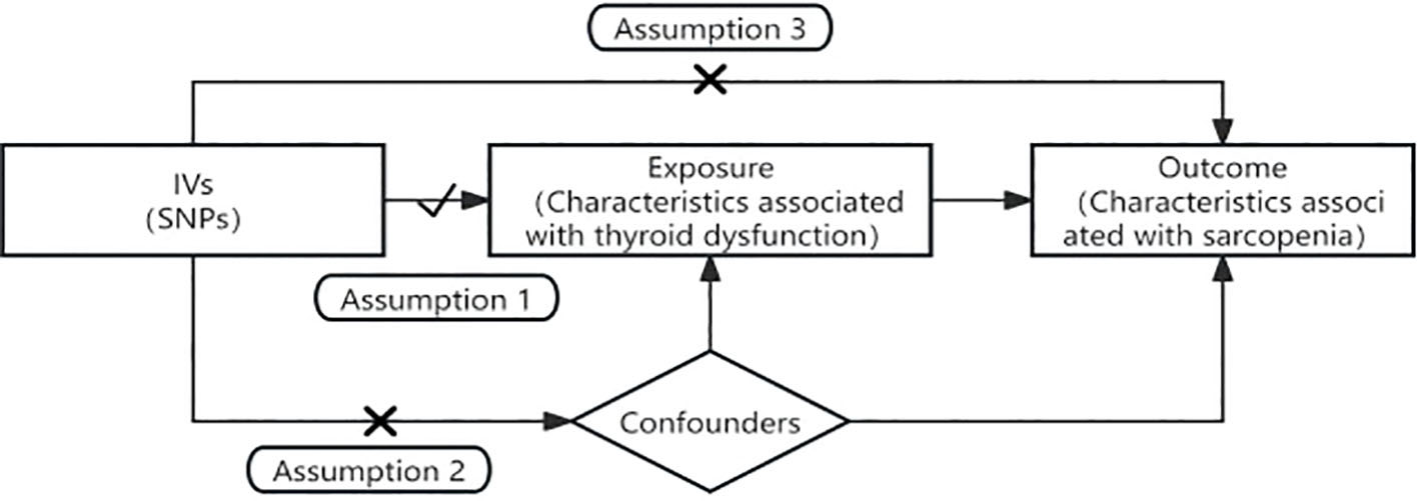
Flowchart and the three core assumptions in Mendelian randomization. Assumption 1: the IVs must be significantly correlated with thyroid dysfunction. Assumption 2: the IVs must be independent of confounders related to both thyroid dysfunction and sarcopenia. Assumption 3: the IVs must affect sarcopenia exclusively through their impact on thyroid dysfunction, with no direct association with sarcopenia.

In this study, hyperthyroidism, hypothyroidism, subclinical hyperthyroidism, and subclinical hypothyroidism were used as exposures. The three sarcopenia-associated features, namely, low grip strength, ALM, and stride speed, were used as outcomes, and two-sample MR was used as the design methodology to explore the causal association between thyroid dysfunction and sarcopenia. In alignment with the STROBE-MR guidelines (14), MR analysis employs IVs to infer causal associations. For the IVs to be valid, the following three core assumptions must be met: 1) The IVs must be significantly correlated with thyroid dysfunction; 2) The IVs must be independent of confounders related to both thyroid dysfunction and sarcopenia; 3) The IVs must affect sarcopenia exclusively through their impact on thyroid dysfunction, with no direct association with sarcopenia. **Figure 1** provides a visual representation of the study’s flow.

### 2.2 Data sources

The SNPs analyzed in this study were sourced from publicly available databases including the FinnGen, the UK Biobank, and the Thyroidomics Consortium. Access to these datasets was facilitated through R and Python packages, which interface with the respective application programming interfaces. All SNPs were extracted from studies involving European populations to mitigate the effects of population stratification. Since the study utilized published genome-wide association studies (GWAS) data, no ethical clearance or informed consent was required. A detailed overview of the GWAS data is shown in **Table 1**.

**Table 1:** The relevant data information was extracted from the GWAS database.

Phenotype	Data source	Years	Sample size	No. of SNPs	GWAS ID
Hyperthyroidism	FinnGen	2021	173,938	16,380,189	finn-b-AUTOIMMUNE_HYPERTHYROIDISM
Hypothyroidism	UK Biobank	2018	462,933	9,851,867	ukb-b-19732
Subclinical hyperthyroidism	Thyroidomics Consortium	2018	51,823	8,048,941	NA
Subclinical hypothyroidism	Thyroidomics Consortium	2018	53,423	8,048,941	NA
Low hand grip strength	UK Biobank	2021	256,523	9,336,415	ebi-a-GCST90007526
ALM	UK Biobank	2020	450,243	18,071,518	ebi-a-GCST90000025
Walking pace	UK Biobank	2018	459,915	9,851,867	ukb-b-4711

ALM, appendicular lean mass; No. of SNPs, number of single nucleotide polymorphisms. NA, Not Available.

Summary GWAS data for hyperthyroidism are derived from the FinnGen study, comprising 173,938 subjects and over 16 million SNPs. Hypothyroidism data was sourced from the UK Biobank, including 462,933 subjects and over 9 million SNPs. Data on subclinical hyperthyroidism and subclinical hypothyroidism were derived from a meta-analysis investigating the genetic basis of thyroid function. The thyroid function is assessed by measuring circulating TSH and free T4 levels involving testing over 8 million genetic variants in a sample of up to 72,167 individuals (15).

The findings on low grip strength were obtained from a comprehensive genome-wide association meta-analysis involving 22 cohorts of 256,523 European individuals aged 60 years or older (16). Of these participants, 46,596 (18.9%) exhibited muscle weakness, as identified through 15 specific genetic loci associated with muscle weakness by the European Working Group on Sarcopenia in Older People. ALM summary data included 450,243 participants measured primarily by the Tanita BC 418ma body fat analyzer, validated against DEXA methodology (17). Walking pace data were derived from a 2018 study including 459,915 cases from a European population, with 9,851,867 SNPs analyzed.

### 2.3 Choice of instrumental variables

For the IVs to be valid, the study followed three fundamental assumptions. Firstly, the IVs must be significantly correlated with the exposure factor (thyroid dysfunction). This was validated by selecting SNPs with a *P* < 5×10^−8^ from the GWAS data. Secondly, the IVs must be independent of any confounding variables. This was analyzed using the linkage disequilibrium (LD) parameter with conditions of r² < 0.001 and a genetic distance of 10,000 kb (18). Thirdly, the IVs should influence the outcome (sarcopenia) only through the exposure (thyroid dysfunction). Selected IVs were further evaluated using the F statistic to assess for weak instrument bias. F > 10 indicates the absence of weak instrument bias, while F < 10 suggests potential bias, needing the exclusion of the respective SNP to avoid skewing the results (19).

### 2.4 Mendelian randomization analysis

The study employed an inverse-variance weighted (IVW) to combine the effect sizes of multiple SNPs, analyzing the causal associations between thyroid dysfunction and sarcopenia (20). This primary analysis was supplemented by weighted median and MR-Egger regression to ensure robustness (21, 22). The MR-Egger intercept was used to detect horizontal pleiotropy, while Cochran’s Q test assessed heterogeneity in SNP effects (23). Leave-one-out was used to assess whether a single SNP had an effect on the MR results. Additionally, MR-PRESSO was used to detect outlier SNPs, thereby reducing bias due to horizontal pleiotropy (24).

## 3 Results

### 3.1 Instrumental variables

After excluding SNPs by criteria such as screening P-values, removal of LD, F statistic screening, and adjustment for allelic status, five SNPs associated with hyperthyroidism, 116 SNPs associated with hypothyroidism, six SNPs associated with subclinical hyperthyroidism, and eight SNPs associated with subclinical hypothyroidism were finally included as IVs from the original GWAS. **Supplementary Tables 1–4** provide detailed information on the IVs. When utilizing these IVs to correlate with the outcomes, we excluded pleiotropic and palindromic SNPs to improve the robustness of the results.

### 3.2 MR analysis of hyperthyroidism and sarcopenia-related features

**Figure 2:**
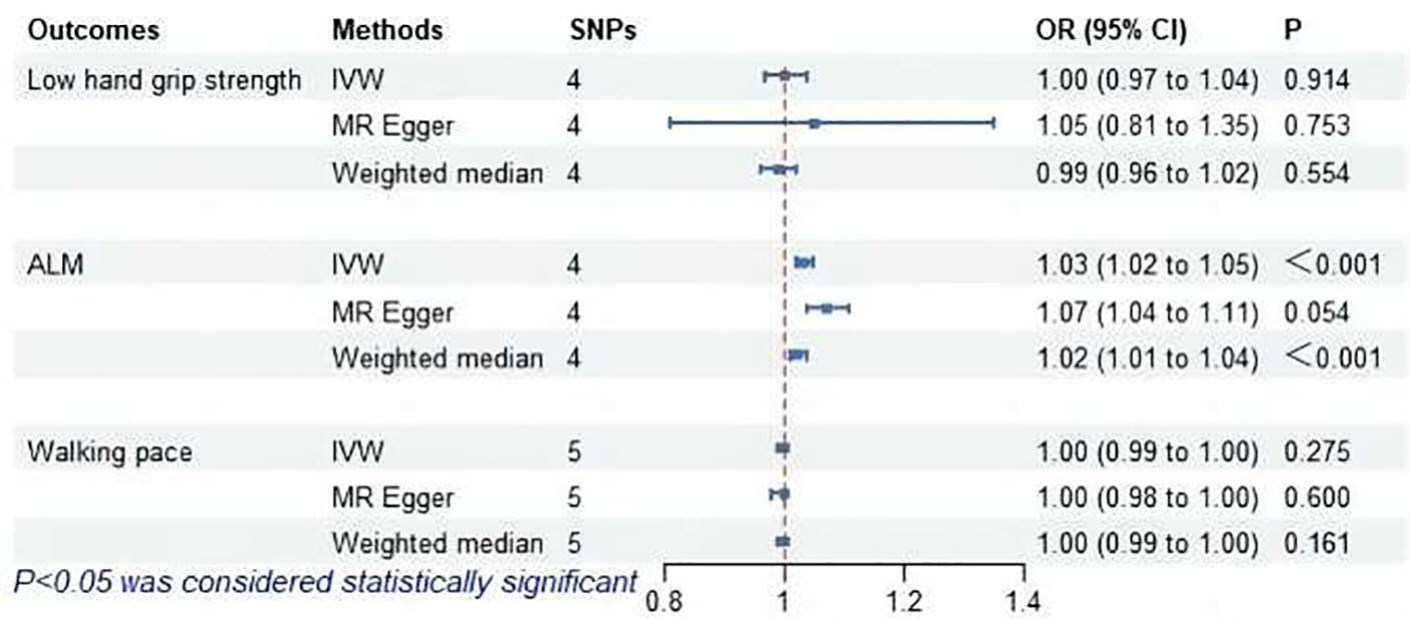
MR analysis of association between hyperthyroidism and sarcopenia-related features. ALM, appendicular lean mass; IVW, inverse-variance weighted; SNPs, single nucleotide polymorphisms; OR, odds ratio; CI, confidence interval.

**Figure 3:**
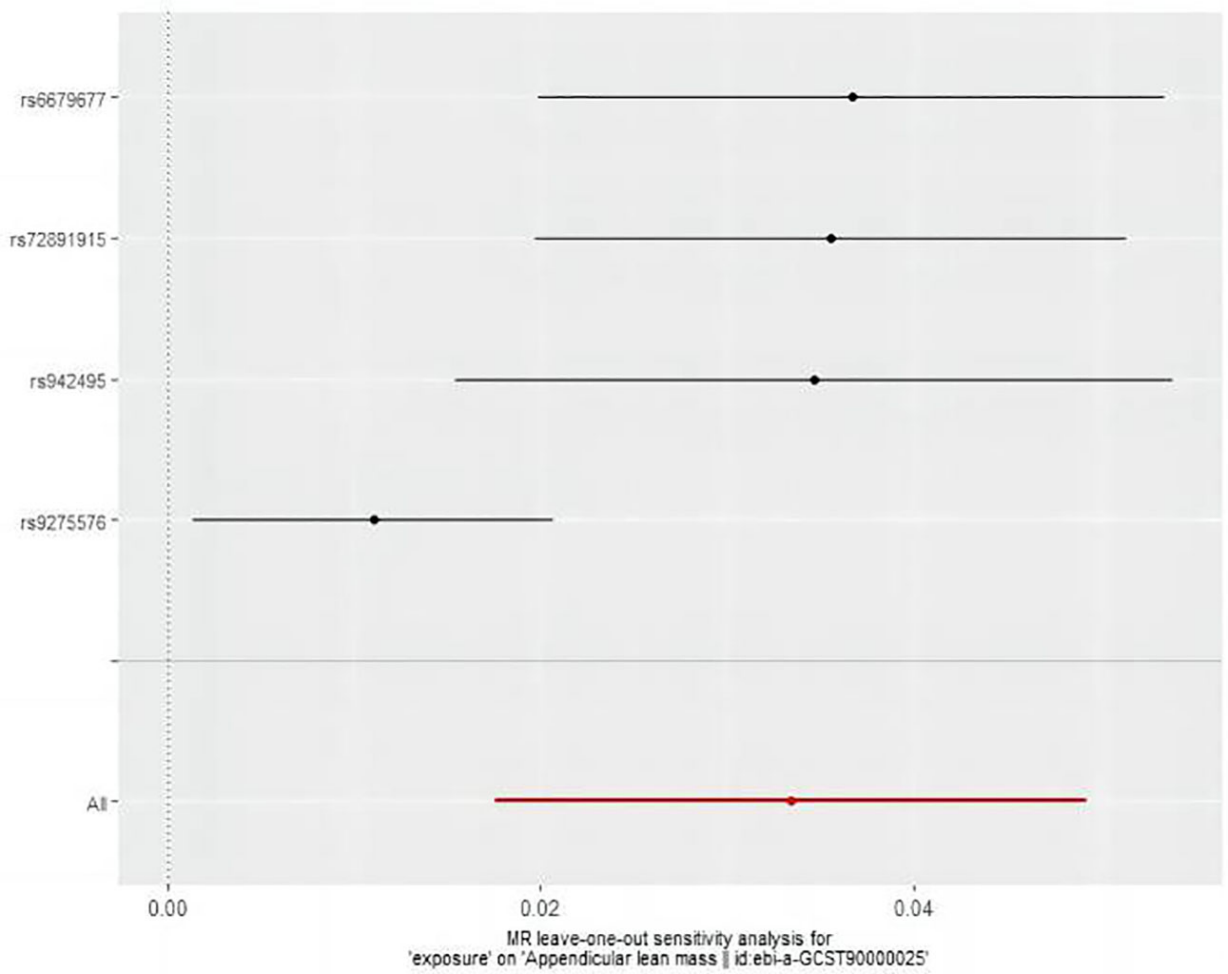
The result of the leave-one-out sensitivity analysis of hyperthyroidism and ALM.

The MR analysis assessed the impact of hyperthyroidism on sarcopenia-related phenotypes. **Figure 2** illustrates these results. The IVW method has shown that hyperthyroidism is associated with reduced ALM (OR = 1.03, 95% CI = 1.02 to 1.05, *P* < 0.001). After removing one outlier SNP (rs179247) using MR-PRESSO, MR-Egger analysis confirmed the absence of pleiotropy (*P* > 0.05). Cochran’s Q test showed heterogeneity among the hyperthyroidism and ALM (*P* < 0.05). Leave-one-out was observed that no significant differences in the results occurred after excluding each SNP, confirming the reliability of the results of the MR analyses. Leave-one-out for hyperthyroidism and ALM are presented in **Figure 3**.

### 3.3 MR analysis of hypothyroidism and sarcopenia-related features

**Figure 4:**
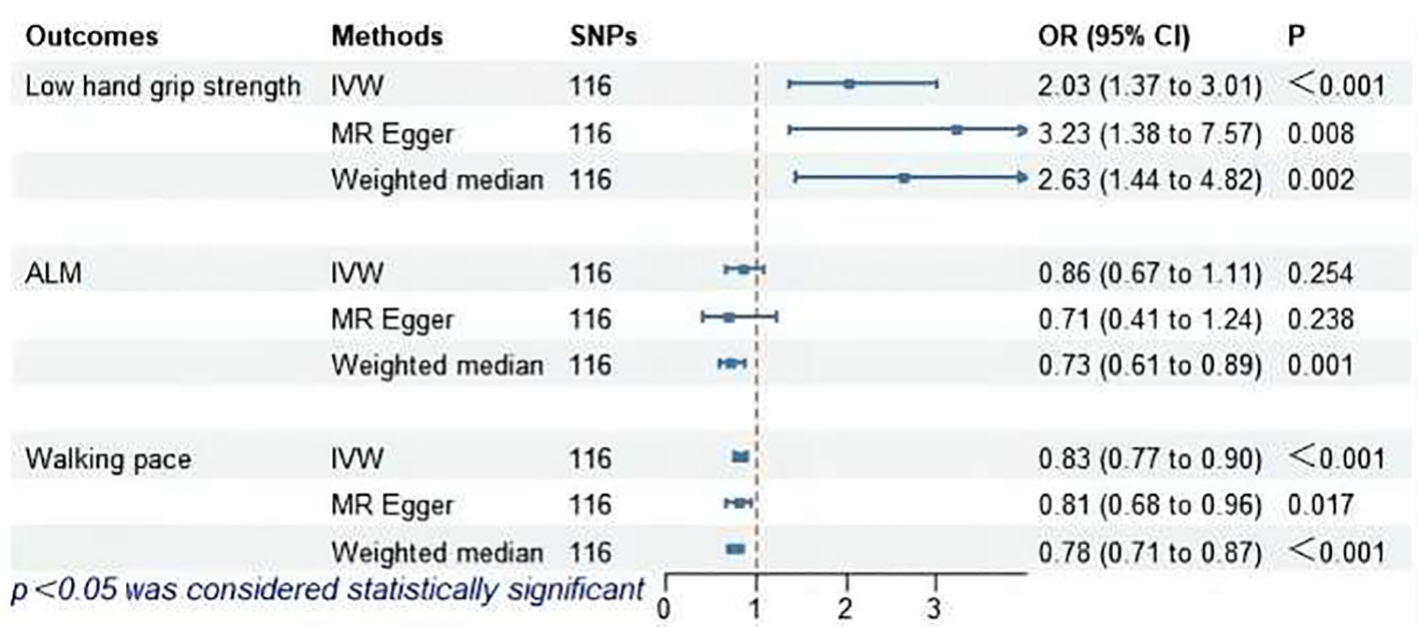
MR analysis of association between hypothyroidism and sarcopenia-related features. ALM, appendicular lean mass; IVW, inverse-variance weighted; SNPs, single nucleotide polymorphisms; OR, odds ratio; CI, confidence interval.

**Figure 5:**
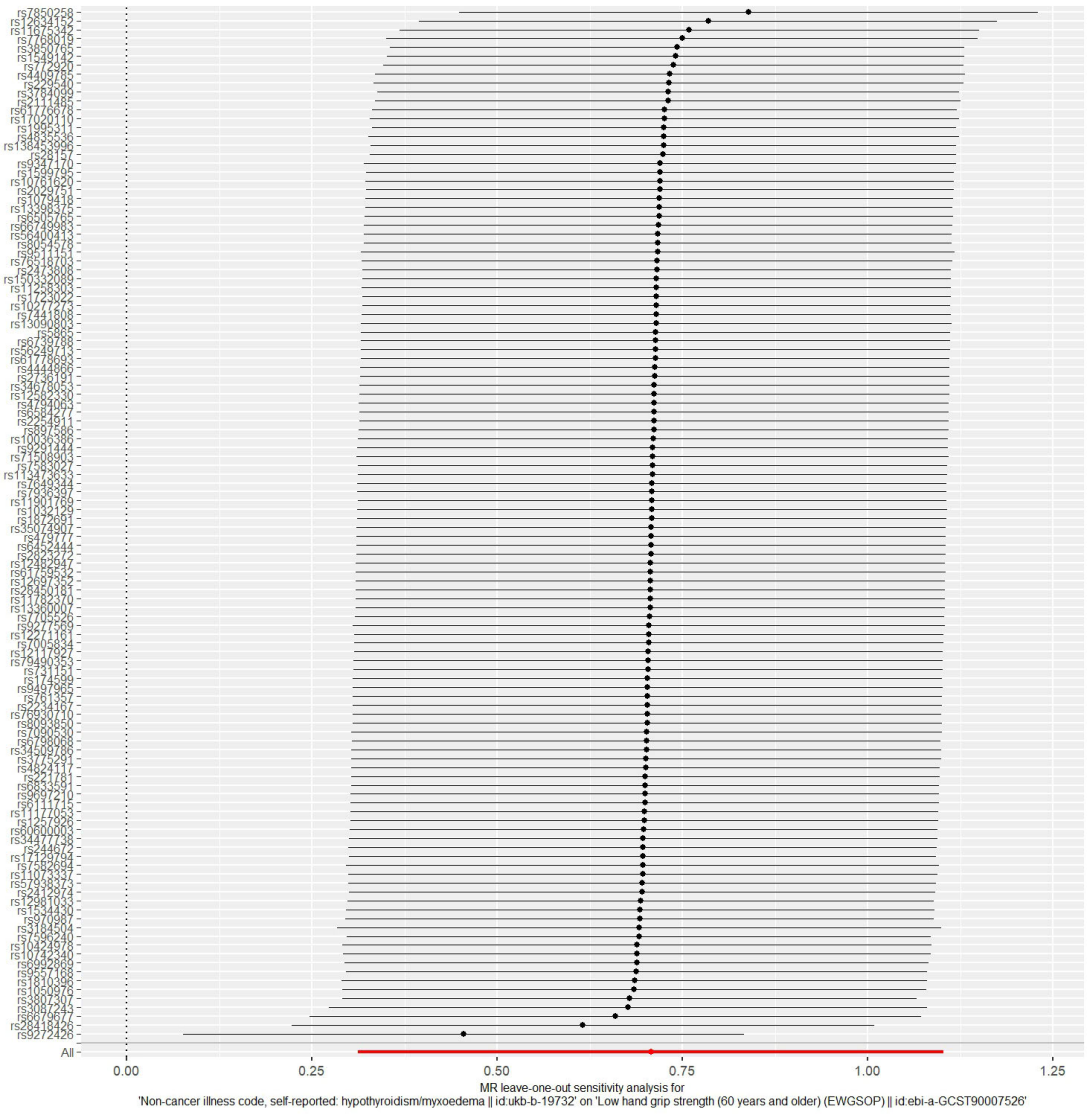
The result of the leave-one-out sensitivity analysis of hypothyroidism and low hand grip strength.

**Figure 6:**
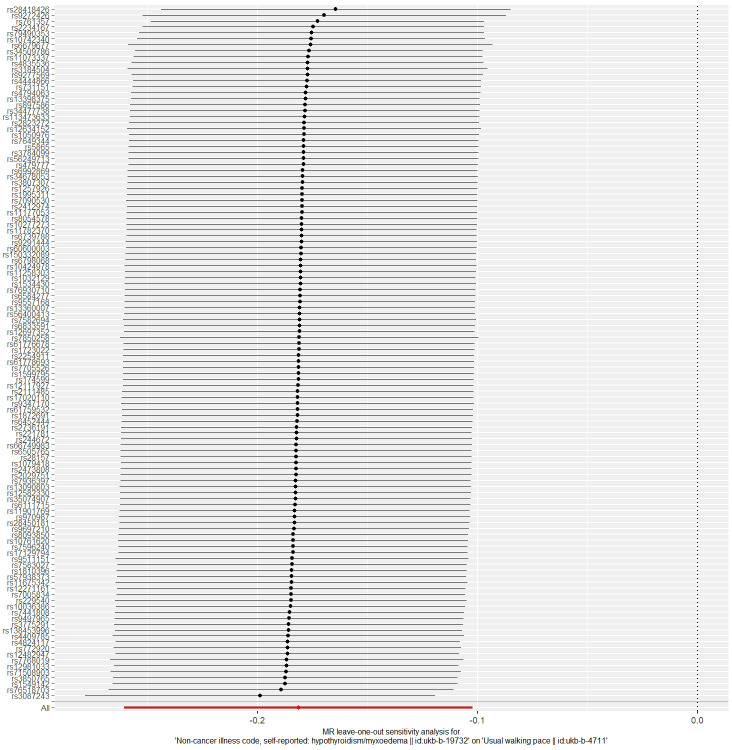
The result of the leave-one-out sensitivity analysis of hypothyroidism and walking pace.

The MR analysis results for hypothyroidism and sarcopenia-related phenotypes are shown in **Figure 4**. The IVW method identified causal associations between hypothyroidism and low hand grip strength (OR = 2.03; 95% CI = 1.37 to 3.01, *P* < 0.001) as well as the causal associations between hypothyroidism and walking pace (OR = 0.83; 95% CI = 0.77 to 0.90, *P* < 0.001). MR-Egger analysis did not detect potential pleiotropy (*P* > 0.05). The results were consistent after correction for abnormal SNPs by MR-PRESSO. Cochran’s Q test indicated heterogeneity among the hypothyroidism and sarcopenia-related features (*P* < 0.05). Leave-one-out indicated no significant changes observed upon sequential SNP exclusion. Leave-one-out for hypothyroidism with low hand grip strength and walking pace are depicted in **Figures 5** and **6**, respectively.

### 3.4 MR analysis of subclinical hyperthyroidism and sarcopenia-related features

**Figure 7:**
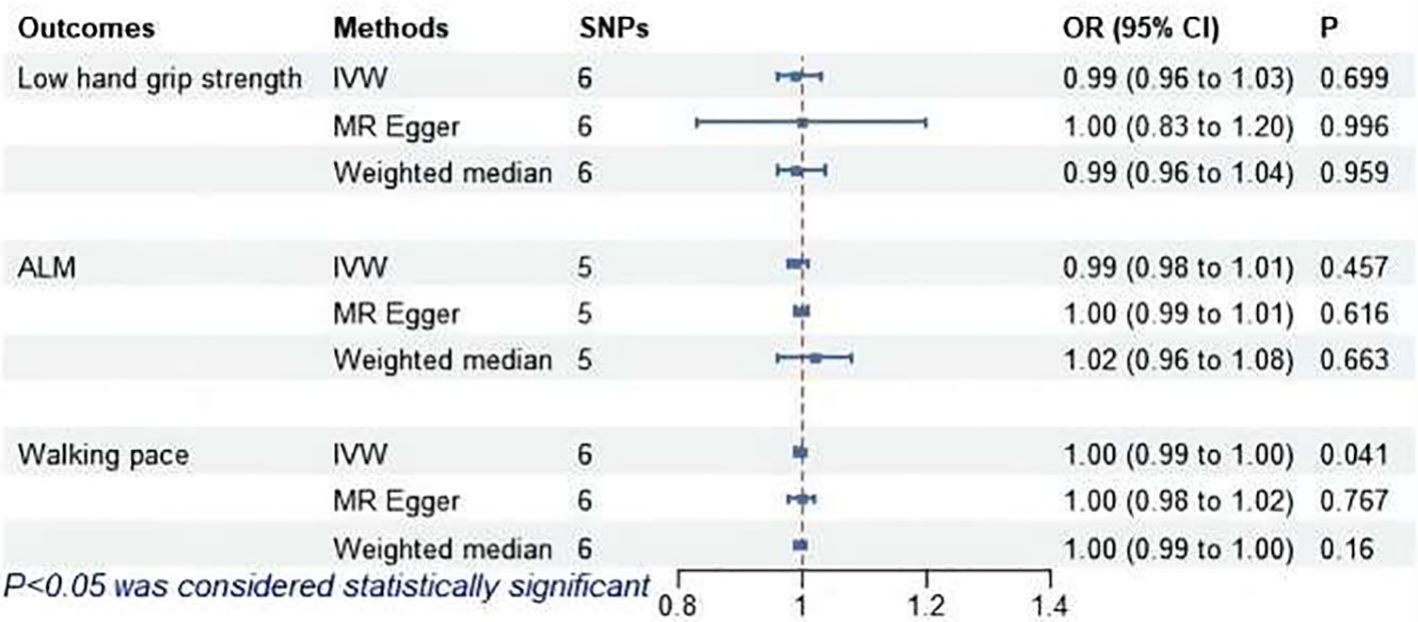
MR analysis of association between subclinical hyperthyroidism and sarcopenia-related features. ALM, appendicular lean mass; IVW, inverse-variance weighted; SNPs, single nucleotide polymorphisms; OR, odds ratio; CI, confidence interval.

**Figure 8:**
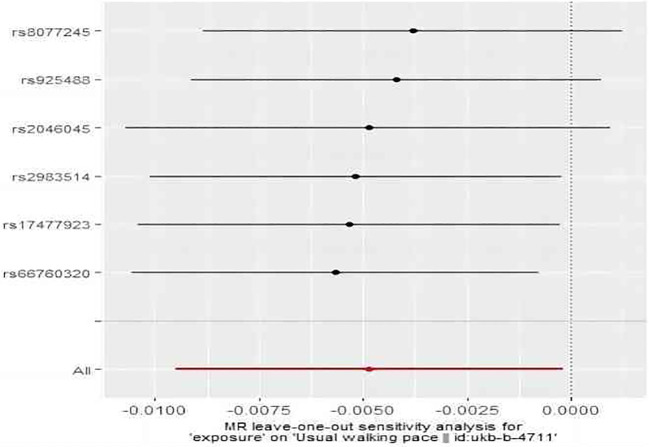
The result of the leave-one-out sensitivity analysis of subclinical hyperthyroidism and walking pace.

As shown in **Figure 7**, IVW revealed a significant association between subclinical hyperthyroidism and decreased walking pace (OR = 1.00; 95% CI = 0.99 to 1.00; *P* = 0.041). MR-Egger regression confirmed the absence of pleiotropy (*P* > 0.05). No outliers were identified by MR-PRESSO. Cochran’s Q test confirmed the presence of heterogeneity (*P* < 0.05). Leave-one-out indicated minimal changes in the overall error line after excluding each SNP, ensuring the robustness of the findings. Leave-one-out for subclinical hyperthyroidism and walking pace are illustrated in **Figure 8**.

### 3.5 MR analysis of subclinical hypothyroidism and sarcopenia-related features

**Figure 9:**
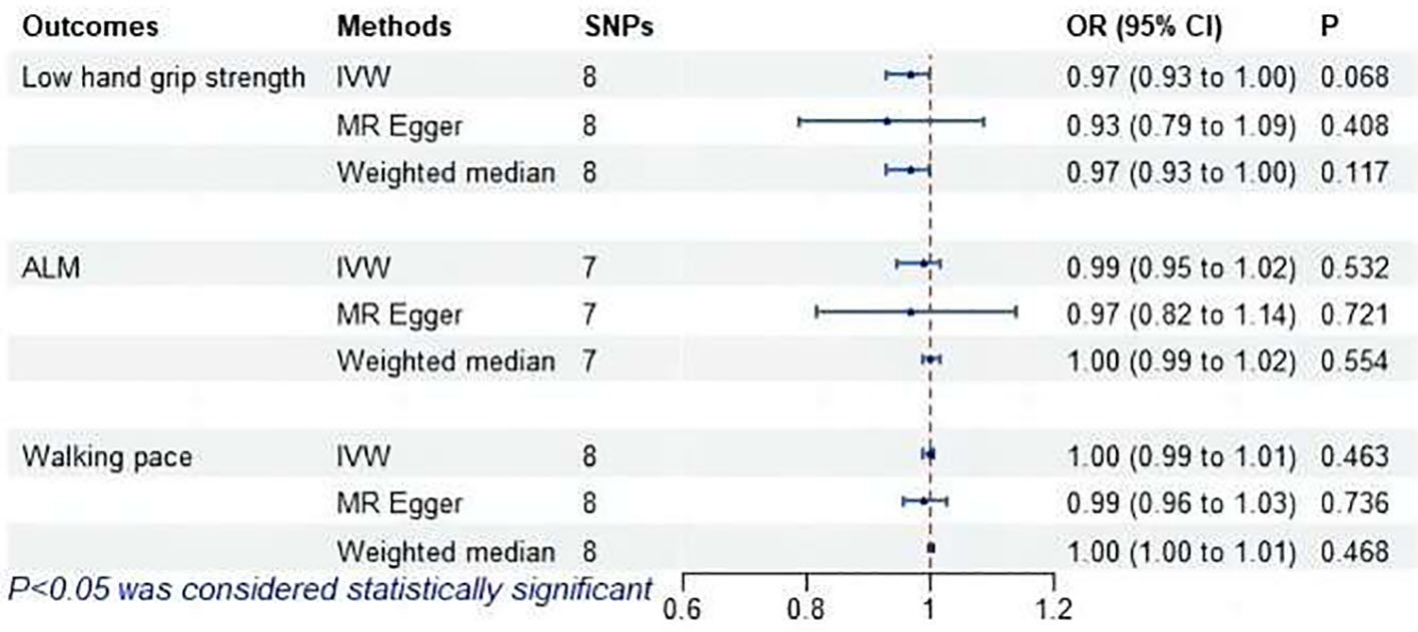
MR analysis of association between subclinical hypothyroidism and sarcopenia-related features. ALM, appendicular lean mass; IVW, inverse-variance weighted; SNPs, single nucleotide polymorphisms; OR, odds ratio; CI, confidence interval.

The MR analysis did not find a causal association between subclinical hypothyroidism and sarcopenia-related features, as shown in **Figure 9**. MR-Egger regression indicated no pleiotropy (*P* > 0.05). No outliers were identified by MR-PRESSO. Cochran’s Q test revealed heterogeneity between subclinical hypothyroidism and both ALM and walking pace (*P* < 0.05). Leave-one-out demonstrated that excluding SNPs one by one had no significant effect on the results, affirming the robustness of the findings.

## 4 Discussion

This study employed a two-sample MR design to systematically explore the causal relationships between thyroid dysfunction and sarcopenia-related features through genetic variation analysis. The findings revealed that hyperthyroidism is associated with reduced muscle mass; hypothyroidism correlates with diminished grip strength and walking pace; subclinical hyperthyroidism is linked to lower walking pace. However, no causal relationship was found between subclinical hypothyroidism and sarcopenia.

Thyroid hormones significantly influence skeletal muscle contraction, repair, and metabolic functions (6, 25, 26). These effects are mediated by thyroid hormone transporters MCT8 and MCT10 in the plasma membrane, the presence of thyroid hormone receptors, and the hormones’ availability (27–29). Emerging clinical evidence highlights the critical role of thyroid function in maintaining muscle health (30, 31). A study by Brennan et al. (32) demonstrated that adults with hyperthyroidism had reduced muscle strength and smaller mid-thigh cross-sectional areas compared to individuals with normal thyroid function. Notably, muscle function improved significantly with the restoration of thyroid function. This finding aligns with our study, which showed hyperthyroidism leads to a reduction in ALM. Studies of thyroid function and grip strength have shown that both hyperthyroidism and hypothyroidism reduce hand grip strength (33, 34). However, our study found a causal association only between hypothyroidism and low hand grip strength. Although previous observational studies did not establish a link between walking pace and thyroid dysfunction, our results demonstrate a causal association between hypothyroidism and walking pace.

Earlier observational studies have investigated the effects of subclinical thyroid dysfunction on muscle function and physical performance. One study on older individuals found that subclinical hypothyroidism minimally affects muscle mass, strength, and quality, suggesting no link to sarcopenia (35). Similarly, a study of elderly Brazilians found no association between subclinical thyroid dysfunction and sarcopenia (8). Contradicting these findings, our study suggests that subclinical hyperthyroidism may decrease walking pace, thereby increasing the risk of sarcopenia. Thyroid disease in the elderly often presents atypically, easily confused with aging symptoms. A cross-sectional study showed that changes in thyroid hormone levels were associated with frailty in older adults, suggesting a relevant role for thyroid function in aging (36). A study on subclinical thyroid dysfunction and frailty in older men indicated that subclinical hyperthyroidism increased the level of frailty (37). Sarcopenia as an independent predictor of all-cause mortality in the elderly population, a study by Zhang (38) et al. found a positive correlation between free triiodothyronine (FT3) and grip strength, suggesting that FT3 is a prognostic determinant of sarcopenia, emphasizing that routine assessment of FT3 is mandatory for identifying patients at risk for sarcopenia.

Our study leveraged stable genetic variants representing specific phenotypes as IVs, effectively mitigating confounding effects and reverse causality, thereby highlighting the lifelong risk of sarcopenia associated with thyroid dysfunction. However, several limitations exist. The study analyzed GWAS data for three sarcopenia-related phenotypes rather than direct sarcopenia GWAS data, limiting a comprehensive assessment. In addition, the GWAS for walking pace was based on an ordered trichotomous phenotype in the UK Biobank, potentially obscuring finer differences in walking pace. Future GWAS should consider walking pace as a continuous variable or use internationally recognized standards like 0.8 m/s to create a binary variable (39). Furthermore, the GWAS data were exclusively from European populations, limiting the applicability to other ethnic groups. Further research is needed with GWAS data from diverse populations of other ethnic groups to explore potential genetic background differences.

In conclusion, this study reveals that hyperthyroidism, hypothyroidism, and subclinical hyperthyroidism may be genetic risk factors for sarcopenia. Recognizing the correlation between thyroid dysfunction and sarcopenia in clinical assessments is crucial for preventing and managing sarcopenia effectively. Integrating thyroid function evaluation into routine sarcopenia screening could enhance early detection and intervention strategies.

## Data Availability

The datasets presented in this study can be found in online repositories. The names of the repository/repositories and accession number(s) can be found in the article/**Supplementary Material**.
