# Supplementary material for: Thyroid dysfunction and sarcopenia: a two-sample Mendelian randomization study
Source: Front Endocrinol (Lausanne). 2024 Sep 5;15:1378757. doi: 10.3389/fendo.2024.1378757 (PMC11410624; doi:10.3389/fendo.2024.1378757)
Supplement: Supplementary file 1 [file Table1.docx]

Supplementary Material

**Supplementary Table 1.** Genome-wide significant SNPs for Hyperthyroidis 1

**Supplementary Table 2.** Genome-wide significant SNPs for Hypothyroidis 1

**Supplementary Table 3.** Genome-wide significant SNPs for Subclinical hyperthyroidism 8

**Supplementary Table 4.** Genome-wide significant SNPs for subclinical hypothyroidism 9

**Supplementary Table 1.** Genome-wide significant SNPs for Hyperthyroidism

| SNP | Outcome | EA | OA | BETA | SE | EAF | P |
| --- | --- | --- | --- | --- | --- | --- | --- |
| rs179247 | Low hand grip strength | G | A | 0.0074 | 0.0074 | 0.4826 | 0.3172 |
| rs6679677 | Low hand grip strength | A | C | 0.0255 | 0.0122 | 0.1002 | 0.0366 |
| rs72891915 | Low hand grip strength | A | G | -0.0208 | 0.0285 | 0.0194 | 0.4661 |
| rs942495 | Low hand grip strength | T | C | -0.0006 | 0.0167 | 0.0566 | 0.9694 |
| rs6679677 | ALM | A | C | 0.0057 | 0.0031 | 0.101 | 0.067671 |
| rs72891915 | ALM | A | G | -0.0009 | 0.0069 | 0.0196 | 0.8985 |
| rs9275576 | ALM | T | C | 0.0397 | 0.0027 | 0.1443 | 1.65806E-49 |
| rs942495 | ALM | T | C | 0.0092 | 0.0044 | 0.051 | 0.03602 |
| rs179247 | Walking pace | G | A | 3.74E-05 | 0.001273 | 0.479878 | 0.98 |
| rs6679677 | Walking pace | A | C | -0.00495 | 0.002098 | 0.100752 | 0.018 |
| rs72891915 | Walking pace | A | G | 0.005957 | 0.004657 | 0.019649 | 0.2 |
| rs9275576 | Walking pace | T | C | -0.00301 | 0.001805 | 0.144195 | 0.096 |
| rs942495 | Walking pace | T | C | 0.002623 | 0.002929 | 0.051397 | 0.37 |

**Supplementary Table 2.** Genome-wide significant SNPs for Hypothyroidism

| SNP | Outcome | EA | OA | BETA | SE | EAF | P |
| --- | --- | --- | --- | --- | --- | --- | --- |
| rs10036386 | Low hand grip strength | T | C | 7.00E-04 | 0.0075 | 0.3859 | 0.9223 |
| rs10277273 | Low hand grip strength | G | T | 0.0034 | 0.0076 | 0.573 | 0.6516 |
| rs1032129 | Low hand grip strength | C | A | -0.001 | 0.0077 | 0.3546 | 0.8983 |
| rs10424978 | Low hand grip strength | A | C | -0.0114 | 0.0076 | 0.5994 | 0.1346 |
| rs1050976 | Low hand grip strength | T | C | 0.0155 | 0.0074 | 0.5165 | 0.03625 |
| rs10742340 | Low hand grip strength | C | T | 0.012 | 0.0076 | 0.3906 | 0.1111 |
| rs10761620 | Low hand grip strength | G | A | 0.0043 | 0.0075 | 0.5508 | 0.5612 |
| rs1079418 | Low hand grip strength | G | A | 0.0061 | 0.0079 | 0.3084 | 0.4416 |
| rs11073337 | Low hand grip strength | C | A | 0.0095 | 0.0085 | 0.2529 | 0.2596 |
| rs11177053 | Low hand grip strength | C | T | -0.0083 | 0.0075 | 0.4045 | 0.2657 |
| rs11258303 | Low hand grip strength | A | C | -0.0024 | 0.0085 | 0.7426 | 0.7755 |
| rs113473633 | Low hand grip strength | G | A | -0.0026 | 0.0255 | 0.0251 | 0.9201 |
| rs11675342 | Low hand grip strength | T | C | -0.0156 | 0.0074 | 0.4249 | 0.03576 |
| rs11782370 | Low hand grip strength | T | C | -0.0029 | 0.0087 | 0.236 | 0.741899 |
| rs11901769 | Low hand grip strength | T | A | 7.00E-04 | 0.0083 | 0.271 | 0.9349 |
| rs12117927 | Low hand grip strength | A | C | 0.0044 | 0.0076 | 0.4883 | 0.5652 |
| rs12271161 | Low hand grip strength | A | G | -0.0048 | 0.0093 | 0.1958 | 0.6051 |
| rs12482947 | Low hand grip strength | C | T | 0.0022 | 0.0076 | 0.6019 | 0.776 |
| rs1257926 | Low hand grip strength | A | G | 0.0082 | 0.0074 | 0.4758 | 0.2651 |
| rs12582330 | Low hand grip strength | T | G | -0.0002 | 0.0083 | 0.7262 | 0.9784 |
| rs12634152 | Low hand grip strength | T | C | 0.0173 | 0.0074 | 0.5436 | 0.01986 |
| rs12697352 | Low hand grip strength | A | G | -0.0023 | 0.0078 | 0.3389 | 0.771699 |
| rs12981033 | Low hand grip strength | G | A | -0.0114 | 0.0076 | 0.3906 | 0.1316 |
| rs13090803 | Low hand grip strength | T | G | -0.0007 | 0.0091 | 0.2106 | 0.9409 |
| rs13360007 | Low hand grip strength | G | A | 0.0039 | 0.0107 | 0.1353 | 0.713099 |
| rs13398375 | Low hand grip strength | C | T | 0.0064 | 0.0081 | 0.2919 | 0.4298 |
| rs138453996 | Low hand grip strength | A | G | -0.0331 | 0.0271 | 0.0199 | 0.2227 |
| rs150332089 | Low hand grip strength | A | C | -0.0057 | 0.0108 | 0.1365 | 0.5968 |
| rs1534430 | Low hand grip strength | T | C | -0.0101 | 0.0075 | 0.3919 | 0.1786 |
| rs1549142 | Low hand grip strength | T | C | -0.0198 | 0.0088 | 0.2303 | 0.0241 |
| rs1599795 | Low hand grip strength | A | T | -0.0065 | 0.0093 | 0.1914 | 0.4837 |
| rs17020110 | Low hand grip strength | C | T | -0.0058 | 0.0083 | 0.2671 | 0.4857 |
| rs17129794 | Low hand grip strength | C | A | 0.013 | 0.0097 | 0.1714 | 0.1825 |
| rs1723022 | Low hand grip strength | T | G | -0.0036 | 0.0077 | 0.3678 | 0.6422 |
| rs174599 | Low hand grip strength | C | G | -0.0051 | 0.0076 | 0.376 | 0.5025 |
| rs1810396 | Low hand grip strength | G | A | -0.0154 | 0.008 | 0.6913 | 0.05362 |
| rs1872691 | Low hand grip strength | A | G | -0.0016 | 0.0095 | 0.1836 | 0.8632 |
| rs1995311 | Low hand grip strength | G | T | 0.0106 | 0.0074 | 0.5122 | 0.1521 |
| rs2029751 | Low hand grip strength | C | A | -0.009 | 0.0114 | 0.1188 | 0.4322 |
| rs2111485 | Low hand grip strength | G | A | -0.009 | 0.0075 | 0.6049 | 0.231 |
| rs221781 | Low hand grip strength | G | A | 0.0111 | 0.0117 | 0.8858 | 0.3434 |
| rs2234167 | Low hand grip strength | A | G | 0.0077 | 0.0108 | 0.1346 | 0.4766 |
| rs2254911 | Low hand grip strength | C | T | 0.0014 | 0.0146 | 0.9291 | 0.9239 |
| rs229540 | Low hand grip strength | G | T | -0.0052 | 0.0074 | 0.4256 | 0.4821 |
| rs2412974 | Low hand grip strength | T | C | -0.0114 | 0.0076 | 0.3615 | 0.134 |
| rs244672 | Low hand grip strength | T | C | -0.0128 | 0.011 | 0.8697 | 0.2455 |
| rs2473808 | Low hand grip strength | C | T | 0.0032 | 0.0078 | 0.6635 | 0.684499 |
| rs2736191 | Low hand grip strength | G | C | 0.0031 | 0.0219 | 0.0314 | 0.8873 |
| rs28157 | Low hand grip strength | T | G | 0.008 | 0.0079 | 0.3127 | 0.3126 |
| rs2823272 | Low hand grip strength | A | T | -0.0022 | 0.008 | 0.3144 | 0.783 |
| rs28418426 | Low hand grip strength | C | T | 0.0307 | 0.0085 | 0.508 | 0.000281 |
| rs28450181 | Low hand grip strength | G | A | 0.0031 | 0.0092 | 0.2017 | 0.737099 |
| rs3087243 | Low hand grip strength | A | G | -0.0126 | 0.0074 | 0.4498 | 0.08739 |
| rs3184504 | Low hand grip strength | C | T | -0.01 | 0.0074 | 0.5182 | 0.1733 |
| rs34477738 | Low hand grip strength | G | A | 0.0119 | 0.0103 | 0.1642 | 0.2464 |
| rs34509786 | Low hand grip strength | G | T | 0.0075 | 0.0095 | 0.1825 | 0.4282 |
| rs34678053 | Low hand grip strength | A | G | 5.00E-04 | 0.0089 | 0.4889 | 0.9566 |
| rs35074907 | Low hand grip strength | A | G | 0.0054 | 0.0273 | 0.0196 | 0.8435 |
| rs3775291 | Low hand grip strength | T | C | -0.0066 | 0.0082 | 0.2965 | 0.4181 |
| rs3784099 | Low hand grip strength | A | G | 0.0143 | 0.0081 | 0.2844 | 0.077711 |
| rs3807307 | Low hand grip strength | C | T | 0.0227 | 0.0074 | 0.4629 | 0.002042 |
| rs3850765 | Low hand grip strength | C | T | -0.0194 | 0.0075 | 0.589 | 0.009721 |
| rs4409785 | Low hand grip strength | C | T | -0.0072 | 0.0097 | 0.1726 | 0.459 |
| rs4444866 | Low hand grip strength | T | C | 0.0014 | 0.0083 | 0.2742 | 0.8613 |
| rs4794063 | Low hand grip strength | T | C | 5.00E-04 | 0.0084 | 0.2608 | 0.952 |
| rs479777 | Low hand grip strength | C | T | -0.0019 | 0.0078 | 0.3423 | 0.8023 |
| rs4824117 | Low hand grip strength | G | A | -0.0077 | 0.0079 | 0.6667 | 0.3268 |
| rs4835536 | Low hand grip strength | T | G | 0.0029 | 0.009 | 0.2109 | 0.750601 |
| rs56249713 | Low hand grip strength | C | T | 0.0018 | 0.0075 | 0.4192 | 0.8059 |
| rs56400413 | Low hand grip strength | A | T | -0.0057 | 0.0081 | 0.2845 | 0.4865 |
| rs57938373 | Low hand grip strength | T | C | 0.0131 | 0.0103 | 0.1482 | 0.2051 |
| rs5865 | Low hand grip strength | T | C | 0.002 | 0.0078 | 0.6635 | 0.7997 |
| rs60600003 | Low hand grip strength | G | T | 0.0143 | 0.0123 | 0.1 | 0.2436 |
| rs6111715 | Low hand grip strength | C | G | -0.0096 | 0.0096 | 0.1807 | 0.3213 |
| rs61759532 | Low hand grip strength | T | C | 0.0032 | 0.0088 | 0.2402 | 0.718899 |
| rs61776678 | Low hand grip strength | A | G | 0.0102 | 0.0075 | 0.4094 | 0.1756 |
| rs61778693 | Low hand grip strength | T | G | 0.0028 | 0.0086 | 0.2431 | 0.7406 |
| rs6452444 | Low hand grip strength | C | T | 0.0019 | 0.0086 | 0.7554 | 0.8241 |
| rs6505765 | Low hand grip strength | G | C | -0.0044 | 0.0078 | 0.3416 | 0.5721 |
| rs6584277 | Low hand grip strength | G | A | 4.00E-04 | 0.0074 | 0.5262 | 0.9553 |
| rs66749983 | Low hand grip strength | T | A | -0.0031 | 0.008 | 0.31 | 0.698499 |
| rs6679677 | Low hand grip strength | A | C | 0.0255 | 0.0122 | 0.1002 | 0.0366 |
| rs6739788 | Low hand grip strength | T | A | -0.006 | 0.0163 | 0.0538 | 0.7136 |
| rs6798068 | Low hand grip strength | A | G | 0.0067 | 0.0079 | 0.3178 | 0.3959 |
| rs6833591 | Low hand grip strength | G | A | -0.0077 | 0.0077 | 0.3449 | 0.3191 |
| rs6992869 | Low hand grip strength | C | T | 0.0152 | 0.0076 | 0.3777 | 0.04492 |
| rs7005834 | Low hand grip strength | T | C | -0.0043 | 0.008 | 0.3053 | 0.591199 |
| rs7090530 | Low hand grip strength | A | C | 0.0055 | 0.0075 | 0.6013 | 0.4626 |
| rs71508903 | Low hand grip strength | T | C | 0.0037 | 0.0094 | 0.1944 | 0.6915 |
| rs731151 | Low hand grip strength | A | G | 0.0059 | 0.0098 | 0.167 | 0.5471 |
| rs7441808 | Low hand grip strength | G | A | -0.0012 | 0.008 | 0.299 | 0.8851 |
| rs7582694 | Low hand grip strength | G | C | -0.0091 | 0.0088 | 0.7731 | 0.3032 |
| rs7583027 | Low hand grip strength | C | A | 6.00E-04 | 0.0077 | 0.6426 | 0.9337 |
| rs7596240 | Low hand grip strength | G | A | 0.0151 | 0.0082 | 0.2707 | 0.06763 |
| rs761357 | Low hand grip strength | T | A | 0.0052 | 0.0076 | 0.376 | 0.494 |
| rs7649344 | Low hand grip strength | C | T | -0.0007 | 0.0074 | 0.4563 | 0.9254 |
| rs76518703 | Low hand grip strength | G | A | 0.0019 | 0.0175 | 0.0504 | 0.9156 |
| rs76930710 | Low hand grip strength | C | T | -0.0147 | 0.0193 | 0.0389 | 0.445 |
| rs7705526 | Low hand grip strength | A | C | -0.0036 | 0.008 | 0.3263 | 0.6561 |
| rs772920 | Low hand grip strength | G | C | -0.0152 | 0.0078 | 0.333 | 0.051451 |
| rs7768019 | Low hand grip strength | G | C | 0.0073 | 0.0085 | 0.2482 | 0.3873 |
| rs7850258 | Low hand grip strength | G | A | -0.0196 | 0.0078 | 0.6691 | 0.01222 |
| rs7936397 | Low hand grip strength | A | G | -0.0008 | 0.0083 | 0.2699 | 0.9205 |
| rs79490353 | Low hand grip strength | C | T | 0.0142 | 0.0236 | 0.0254 | 0.547599 |
| rs8054578 | Low hand grip strength | G | A | 0.0048 | 0.0088 | 0.7729 | 0.590799 |
| rs8093850 | Low hand grip strength | G | A | 0.0059 | 0.0079 | 0.3151 | 0.4599 |
| rs897586 | Low hand grip strength | A | G | -0.0003 | 0.0077 | 0.3561 | 0.9707 |
| rs9272426 | Low hand grip strength | G | A | 0.0461 | 0.0076 | 0.4462 | 1.37E-09 |
| rs9277569 | Low hand grip strength | T | C | 0.0074 | 0.0117 | 0.112 | 0.528799 |
| rs9291444 | Low hand grip strength | T | C | 0.0015 | 0.0074 | 0.4691 | 0.8405 |
| rs9347170 | Low hand grip strength | T | C | 6.00E-04 | 0.0078 | 0.3369 | 0.9377 |
| rs9497965 | Low hand grip strength | T | C | 0.005 | 0.0075 | 0.4087 | 0.5029 |
| rs9511151 | Low hand grip strength | A | G | -0.0014 | 0.0078 | 0.3441 | 0.8575 |
| rs9557168 | Low hand grip strength | A | G | -0.0206 | 0.0092 | 0.2056 | 0.02474 |
| rs9697210 | Low hand grip strength | A | G | -0.0113 | 0.0106 | 0.1451 | 0.2856 |
| rs10036386 | ALM | T | C | -0.0008 | 0.0019 | 0.3818 | 0.670199 |
| rs10277273 | ALM | G | T | -0.0106 | 0.0019 | 0.5709 | 3.57E-08 |
| rs1032129 | ALM | C | A | -0.0056 | 0.002 | 0.3556 | 0.004341 |
| rs10424978 | ALM | A | C | 0.0029 | 0.002 | 0.6001 | 0.1382 |
| rs1050976 | ALM | T | C | -0.0035 | 0.0019 | 0.529 | 0.06146 |
| rs10742340 | ALM | C | T | -0.0095 | 0.002 | 0.3877 | 9.91E-07 |
| rs10761620 | ALM | G | A | 0.0033 | 0.0019 | 0.549 | 0.083401 |
| rs1079418 | ALM | G | A | -0.0001 | 0.002 | 0.3064 | 0.9662 |
| rs11073337 | ALM | C | A | -0.0031 | 0.0022 | 0.2518 | 0.158 |
| rs11177053 | ALM | C | T | 0.0011 | 0.0019 | 0.406 | 0.5592 |
| rs11258303 | ALM | A | C | -0.0007 | 0.0022 | 0.7463 | 0.732501 |
| rs113473633 | ALM | G | A | -0.0004 | 0.0062 | 0.026 | 0.9532 |
| rs11675342 | ALM | T | C | 8.00E-04 | 0.0019 | 0.4233 | 0.6611 |
| rs11782370 | ALM | T | C | 2.00E-04 | 0.0022 | 0.2394 | 0.9197 |
| rs11901769 | ALM | T | A | -0.0003 | 0.0021 | 0.2698 | 0.8985 |
| rs12117927 | ALM | A | C | 3.00E-04 | 0.0019 | 0.4888 | 0.8601 |
| rs12271161 | ALM | A | G | -0.0135 | 0.0024 | 0.1933 | 1.76E-08 |
| rs12482947 | ALM | C | T | -0.0019 | 0.002 | 0.6006 | 0.3254 |
| rs1257926 | ALM | A | G | -0.0033 | 0.0019 | 0.4751 | 0.08692 |
| rs12582330 | ALM | T | G | 0.0099 | 0.0021 | 0.7281 | 3.06E-06 |
| rs12634152 | ALM | T | C | 0.0019 | 0.0019 | 0.5472 | 0.3118 |
| rs12697352 | ALM | A | G | 0.0087 | 0.002 | 0.3403 | 1.20E-05 |
| rs12981033 | ALM | G | A | -0.0002 | 0.0019 | 0.393 | 0.918 |
| rs13090803 | ALM | T | G | -0.0015 | 0.0023 | 0.2123 | 0.5061 |
| rs13360007 | ALM | G | A | 0.0032 | 0.0028 | 0.1356 | 0.2396 |
| rs13398375 | ALM | C | T | -0.0019 | 0.0021 | 0.2922 | 0.361 |
| rs138453996 | ALM | A | G | 0.0163 | 0.0068 | 0.0201 | 0.01575 |
| rs150332089 | ALM | A | C | 0.0028 | 0.0027 | 0.1373 | 0.3046 |
| rs1534430 | ALM | T | C | 0.002 | 0.0019 | 0.3903 | 0.3106 |
| rs1549142 | ALM | T | C | 0.0067 | 0.0023 | 0.2279 | 0.003273 |
| rs1599795 | ALM | A | T | -0.0006 | 0.0024 | 0.1916 | 0.804 |
| rs17020110 | ALM | C | T | -0.0032 | 0.0021 | 0.2674 | 0.1268 |
| rs17129794 | ALM | C | A | 0.001 | 0.0025 | 0.1694 | 0.6814 |
| rs1723022 | ALM | T | G | 0.0051 | 0.002 | 0.3727 | 0.009562 |
| rs174599 | ALM | C | G | -0.0117 | 0.002 | 0.3759 | 2.34E-09 |
| rs1810396 | ALM | G | A | 0.0073 | 0.002 | 0.6893 | 0.000324 |
| rs1872691 | ALM | A | G | -0.0034 | 0.0025 | 0.182 | 0.161 |
| rs1995311 | ALM | G | T | 0.002 | 0.0019 | 0.511 | 0.2874 |
| rs2029751 | ALM | C | A | -0.002 | 0.0029 | 0.1204 | 0.4785 |
| rs2111485 | ALM | G | A | -0.0014 | 0.0019 | 0.6073 | 0.4497 |
| rs221781 | ALM | G | A | 0.0103 | 0.003 | 0.8868 | 0.000505 |
| rs2234167 | ALM | A | G | 0.0071 | 0.0028 | 0.1334 | 0.01008 |
| rs2254911 | ALM | C | T | -0.0072 | 0.0037 | 0.9301 | 0.051191 |
| rs229540 | ALM | G | T | 0.0014 | 0.0019 | 0.4251 | 0.4645 |
| rs2412974 | ALM | T | C | 0.018 | 0.002 | 0.36 | 1.53E-19 |
| rs244672 | ALM | T | C | -0.0056 | 0.0029 | 0.8756 | 0.04788 |
| rs2473808 | ALM | C | T | 0.0028 | 0.002 | 0.6632 | 0.1556 |
| rs2736191 | ALM | G | C | 0.0038 | 0.006 | 0.0255 | 0.5187 |
| rs28157 | ALM | T | G | 0.0026 | 0.002 | 0.3153 | 0.1942 |
| rs2823272 | ALM | A | T | 0.0074 | 0.0021 | 0.3155 | 0.000293 |
| rs28418426 | ALM | C | T | -0.0028 | 0.0021 | 0.5292 | 0.1796 |
| rs28450181 | ALM | G | A | 0.0065 | 0.0023 | 0.204 | 0.005535 |
| rs3087243 | ALM | A | G | 0.0044 | 0.0019 | 0.4505 | 0.01813 |
| rs3184504 | ALM | C | T | 0.0183 | 0.0019 | 0.517 | 2.71E-22 |
| rs34477738 | ALM | G | A | -0.0066 | 0.0027 | 0.1644 | 0.01234 |
| rs34509786 | ALM | G | T | -0.0016 | 0.0024 | 0.1819 | 0.5107 |
| rs34678053 | ALM | A | G | 0.002 | 0.0022 | 0.4896 | 0.3594 |
| rs35074907 | ALM | A | G | -0.0293 | 0.0068 | 0.0199 | 1.59E-05 |
| rs3775291 | ALM | T | C | -0.0018 | 0.0021 | 0.2973 | 0.3749 |
| rs3784099 | ALM | A | G | -0.0083 | 0.0021 | 0.2813 | 8.22E-05 |
| rs3807307 | ALM | C | T | -0.0059 | 0.0019 | 0.4643 | 0.001702 |
| rs3850765 | ALM | C | T | 0.0202 | 0.0019 | 0.5859 | 6.24E-26 |
| rs4409785 | ALM | C | T | -0.0053 | 0.0025 | 0.1726 | 0.03514 |
| rs4444866 | ALM | T | C | 0.0034 | 0.0021 | 0.2766 | 0.1088 |
| rs4794063 | ALM | T | C | 0.0052 | 0.0022 | 0.2568 | 0.01644 |
| rs479777 | ALM | C | T | 7.00E-04 | 0.002 | 0.3418 | 0.716699 |
| rs4824117 | ALM | G | A | 0.0053 | 0.002 | 0.6667 | 0.008898 |
| rs4835536 | ALM | T | G | 0.004 | 0.0023 | 0.2124 | 0.082361 |
| rs56249713 | ALM | C | T | 0.0039 | 0.0019 | 0.4193 | 0.0417 |
| rs56400413 | ALM | A | T | -0.0012 | 0.0021 | 0.2868 | 0.568801 |
| rs57938373 | ALM | T | C | -0.0035 | 0.0027 | 0.147 | 0.1939 |
| rs5865 | ALM | T | C | -0.0025 | 0.002 | 0.6646 | 0.2079 |
| rs60600003 | ALM | G | T | -0.0054 | 0.0032 | 0.1003 | 0.08662 |
| rs6111715 | ALM | C | G | 0.0012 | 0.0025 | 0.1796 | 0.6386 |
| rs61759532 | ALM | T | C | -0.0066 | 0.0023 | 0.2468 | 0.003185 |
| rs61776678 | ALM | A | G | -0.012 | 0.0019 | 0.4107 | 2.90E-10 |
| rs61778693 | ALM | T | G | 0.004 | 0.0022 | 0.2468 | 0.06437 |
| rs6452444 | ALM | C | T | 0.0156 | 0.0022 | 0.7577 | 1.55E-12 |
| rs6505765 | ALM | G | C | 0.0038 | 0.002 | 0.344 | 0.06017 |
| rs6584277 | ALM | G | A | -0.0004 | 0.0019 | 0.5249 | 0.8266 |
| rs66749983 | ALM | T | A | -0.0049 | 0.0021 | 0.3107 | 0.01682 |
| rs6679677 | ALM | A | C | 0.0057 | 0.0031 | 0.101 | 0.067671 |
| rs6739788 | ALM | T | A | 0.0017 | 0.0042 | 0.0536 | 0.676901 |
| rs6798068 | ALM | A | G | -0.0002 | 0.002 | 0.3223 | 0.9164 |
| rs6833591 | ALM | G | A | 0.0012 | 0.002 | 0.3471 | 0.533801 |
| rs6992869 | ALM | C | T | -0.0004 | 0.002 | 0.3698 | 0.836 |
| rs7005834 | ALM | T | C | 0.0034 | 0.002 | 0.3075 | 0.098109 |
| rs7090530 | ALM | A | C | 0.0039 | 0.0019 | 0.6024 | 0.04302 |
| rs71508903 | ALM | T | C | -0.0147 | 0.0024 | 0.1941 | 9.78E-10 |
| rs731151 | ALM | A | G | 0.0082 | 0.0025 | 0.1652 | 0.001318 |
| rs7441808 | ALM | G | A | 0.0059 | 0.0021 | 0.3011 | 0.003995 |
| rs7582694 | ALM | G | C | -0.0053 | 0.0022 | 0.774 | 0.01751 |
| rs7583027 | ALM | C | A | -0.0012 | 0.002 | 0.641 | 0.537 |
| rs7596240 | ALM | G | A | -0.0044 | 0.0021 | 0.2701 | 0.03904 |
| rs761357 | ALM | T | A | 0.0022 | 0.002 | 0.3755 | 0.2594 |
| rs7649344 | ALM | C | T | -0.0041 | 0.0019 | 0.4572 | 0.03033 |
| rs76518703 | ALM | G | A | -0.014 | 0.0044 | 0.0522 | 0.001552 |
| rs76930710 | ALM | C | T | -0.0061 | 0.0049 | 0.0395 | 0.2088 |
| rs7705526 | ALM | A | C | 0.0031 | 0.002 | 0.3262 | 0.1273 |
| rs772920 | ALM | G | C | -0.0126 | 0.002 | 0.3358 | 2.61E-10 |
| rs7768019 | ALM | G | C | -0.0134 | 0.0022 | 0.244 | 8.12E-10 |
| rs7850258 | ALM | G | A | -0.0073 | 0.002 | 0.6681 | 0.000278 |
| rs7936397 | ALM | A | G | -0.0039 | 0.0021 | 0.2726 | 0.06603 |
| rs79490353 | ALM | C | T | -0.0136 | 0.006 | 0.0253 | 0.02393 |
| rs8054578 | ALM | G | A | -0.0005 | 0.0023 | 0.7748 | 0.8405 |
| rs8093850 | ALM | G | A | -0.0147 | 0.002 | 0.3175 | 7.14E-13 |
| rs897586 | ALM | A | G | 0.0018 | 0.002 | 0.3573 | 0.3746 |
| rs9272426 | ALM | G | A | -0.006 | 0.0019 | 0.4521 | 0.001753 |
| rs9277569 | ALM | T | C | 0.0134 | 0.003 | 0.1092 | 8.51E-06 |
| rs9291444 | ALM | T | C | 4.00E-04 | 0.0019 | 0.4706 | 0.8227 |
| rs9347170 | ALM | T | C | -0.0042 | 0.002 | 0.3363 | 0.03608 |
| rs9497965 | ALM | T | C | 0.0012 | 0.0019 | 0.4094 | 0.5251 |
| rs9511151 | ALM | A | G | -0.0024 | 0.002 | 0.346 | 0.2339 |
| rs9557168 | ALM | A | G | 3.00E-04 | 0.0024 | 0.2018 | 0.8845 |
| rs9697210 | ALM | A | G | -0.0011 | 0.0027 | 0.1459 | 0.6886 |
| rs970987 | ALM | A | C | -0.0005 | 0.002 | 0.6632 | 0.7964 |
| rs10036386 | Walking pace | T | C | 0.001616 | 0.001303 | 0.381788 | 0.21 |
| rs10277273 | Walking pace | G | T | 0.001548 | 0.001294 | 0.570821 | 0.23 |
| rs1032129 | Walking pace | C | A | 0.001138 | 0.001326 | 0.355691 | 0.39 |
| rs10424978 | Walking pace | A | C | 0.001256 | 0.001307 | 0.600181 | 0.34 |
| rs1050976 | Walking pace | T | C | -0.00205 | 0.001267 | 0.528728 | 0.11 |
| rs10742340 | Walking pace | C | T | -0.00353 | 0.001305 | 0.387773 | 0.0069 |
| rs10761620 | Walking pace | G | A | -0.00073 | 0.001278 | 0.548829 | 0.57 |
| rs1079418 | Walking pace | G | A | -0.00029 | 0.001374 | 0.306764 | 0.83 |
| rs11073337 | Walking pace | C | A | -0.00332 | 0.001463 | 0.251915 | 0.023 |
| rs11177053 | Walking pace | C | T | 0.001631 | 0.001289 | 0.405732 | 0.21 |
| rs11258303 | Walking pace | A | C | -0.00127 | 0.001458 | 0.745987 | 0.38 |
| rs113473633 | Walking pace | G | A | 0.007588 | 0.004145 | 0.025916 | 0.066999 |
| rs11675342 | Walking pace | T | C | 0.000294 | 0.001282 | 0.423556 | 0.82 |
| rs11782370 | Walking pace | T | C | 0.001807 | 0.001486 | 0.2394 | 0.22 |
| rs11901769 | Walking pace | T | A | 0.000915 | 0.001434 | 0.269739 | 0.52 |
| rs12117927 | Walking pace | A | C | -0.00044 | 0.001299 | 0.488877 | 0.74 |
| rs12271161 | Walking pace | A | G | -0.00209 | 0.001603 | 0.193678 | 0.19 |
| rs12482947 | Walking pace | C | T | 0.003308 | 0.001301 | 0.600751 | 0.011 |
| rs1257926 | Walking pace | A | G | -0.00167 | 0.001271 | 0.47509 | 0.19 |
| rs12582330 | Walking pace | T | G | -0.0000997886 | 0.001426 | 0.727767 | 0.94 |
| rs12634152 | Walking pace | T | C | 0.001913 | 0.001274 | 0.547256 | 0.13 |
| rs12697352 | Walking pace | A | G | 0.000782 | 0.001337 | 0.339787 | 0.56 |
| rs12981033 | Walking pace | G | A | -0.00308 | 0.001298 | 0.393286 | 0.018 |
| rs13090803 | Walking pace | T | G | -0.0000812204 | 0.00156 | 0.212241 | 0.96 |
| rs13360007 | Walking pace | G | A | -0.00132 | 0.001848 | 0.135735 | 0.48 |
| rs13398375 | Walking pace | C | T | 0.003018 | 0.001402 | 0.291759 | 0.031 |
| rs138453996 | Walking pace | A | G | 0.008287 | 0.004515 | 0.020166 | 0.065999 |
| rs150332089 | Walking pace | A | C | -0.00183 | 0.001847 | 0.137206 | 0.32 |
| rs1534430 | Walking pace | T | C | 0.001125 | 0.001299 | 0.390405 | 0.39 |
| rs1549142 | Walking pace | T | C | 0.003486 | 0.001515 | 0.227627 | 0.021 |
| rs1599795 | Walking pace | A | T | -0.0007 | 0.001607 | 0.191618 | 0.66 |
| rs17020110 | Walking pace | C | T | -0.00065 | 0.001435 | 0.267471 | 0.649999 |
| rs17129794 | Walking pace | C | A | 0.001886 | 0.001682 | 0.169737 | 0.26 |
| rs1723022 | Walking pace | T | G | -0.00064 | 0.001321 | 0.372749 | 0.630001 |
| rs174599 | Walking pace | C | G | 0.0005 | 0.001309 | 0.375852 | 0.7 |
| rs1810396 | Walking pace | G | A | -0.00107 | 0.001367 | 0.689167 | 0.44 |
| rs1872691 | Walking pace | A | G | 0.00024 | 0.001643 | 0.182183 | 0.88 |
| rs1995311 | Walking pace | G | T | 0.001688 | 0.001268 | 0.511065 | 0.18 |
| rs2029751 | Walking pace | C | A | 0.000302 | 0.001948 | 0.120278 | 0.88 |
| rs2111485 | Walking pace | G | A | -0.0005 | 0.001294 | 0.60676 | 0.7 |
| rs221781 | Walking pace | G | A | 0.000221 | 0.001996 | 0.886988 | 0.91 |
| rs2234167 | Walking pace | A | G | -0.0071 | 0.001858 | 0.133622 | 0.00013 |
| rs2254911 | Walking pace | C | T | 0.001122 | 0.002479 | 0.93009 | 0.649999 |
| rs229540 | Walking pace | G | T | 0.000373 | 0.001282 | 0.425464 | 0.77 |
| rs2412974 | Walking pace | T | C | 0.001737 | 0.001319 | 0.360518 | 0.19 |
| rs244672 | Walking pace | T | C | 2.86E-05 | 0.001915 | 0.875211 | 0.99 |
| rs2473808 | Walking pace | C | T | -0.00018 | 0.001343 | 0.663293 | 0.89 |
| rs2736191 | Walking pace | G | C | -0.00021 | 0.003992 | 0.025669 | 0.96 |
| rs28157 | Walking pace | T | G | -0.0000910418 | 0.001365 | 0.31522 | 0.95 |
| rs2823272 | Walking pace | A | T | 0.002309 | 0.001366 | 0.315466 | 0.091 |
| rs28418426 | Walking pace | C | T | -0.00572 | 0.001391 | 0.528845 | 3.90E-05 |
| rs28450181 | Walking pace | G | A | 0.000988 | 0.001575 | 0.203914 | 0.53 |
| rs3087243 | Walking pace | A | G | -0.00218 | 0.001271 | 0.450712 | 0.086 |
| rs3184504 | Walking pace | C | T | 0.002551 | 0.001265 | 0.517196 | 0.044 |
| rs34477738 | Walking pace | G | A | -0.0033 | 0.001777 | 0.164246 | 0.063 |
| rs34509786 | Walking pace | G | T | -0.00426 | 0.001643 | 0.181547 | 0.0094 |
| rs34678053 | Walking pace | A | G | 0.001912 | 0.001438 | 0.469817 | 0.18 |
| rs35074907 | Walking pace | A | G | 0.002118 | 0.004515 | 0.020007 | 0.64 |
| rs3775291 | Walking pace | T | C | -0.00173 | 0.001384 | 0.297094 | 0.21 |
| rs3784099 | Walking pace | A | G | 0.002208 | 0.001406 | 0.281731 | 0.12 |
| rs3807307 | Walking pace | C | T | -0.00173 | 0.001268 | 0.464499 | 0.17 |
| rs3850765 | Walking pace | C | T | 0.003131 | 0.001285 | 0.586204 | 0.015 |
| rs4409785 | Walking pace | C | T | 0.000979 | 0.001675 | 0.172613 | 0.56 |
| rs4444866 | Walking pace | T | C | 0.003404 | 0.001424 | 0.27619 | 0.017 |
| rs4794063 | Walking pace | T | C | -0.00277 | 0.001448 | 0.257016 | 0.056 |
| rs479777 | Walking pace | C | T | 0.002086 | 0.001337 | 0.341604 | 0.12 |
| rs4824117 | Walking pace | G | A | -0.00331 | 0.001346 | 0.666702 | 0.014 |
| rs4835536 | Walking pace | T | G | 0.002954 | 0.001548 | 0.212618 | 0.056 |
| rs56249713 | Walking pace | C | T | 0.002033 | 0.001291 | 0.418702 | 0.12 |
| rs56400413 | Walking pace | A | T | -0.00092 | 0.0014 | 0.286505 | 0.51 |
| rs57938373 | Walking pace | T | C | 0.002123 | 0.001786 | 0.14701 | 0.23 |
| rs5865 | Walking pace | T | C | 0.002117 | 0.001341 | 0.664053 | 0.11 |
| rs60600003 | Walking pace | G | T | -0.00231 | 0.00212 | 0.100193 | 0.28 |
| rs6111715 | Walking pace | C | G | -0.0006 | 0.00165 | 0.179576 | 0.709999 |
| rs61759532 | Walking pace | T | C | -0.00041 | 0.001509 | 0.246421 | 0.780001 |
| rs61776678 | Walking pace | A | G | 0.000703 | 0.001288 | 0.41067 | 0.59 |
| rs61778693 | Walking pace | T | G | 0.000613 | 0.00147 | 0.246714 | 0.68 |
| rs6452444 | Walking pace | C | T | 3.69E-05 | 0.001484 | 0.757513 | 0.98 |
| rs6505765 | Walking pace | G | C | 3.81E-05 | 0.001333 | 0.343618 | 0.98 |
| rs6584277 | Walking pace | G | A | 0.000987 | 0.001267 | 0.524853 | 0.44 |
| rs66749983 | Walking pace | T | A | -0.0000829086 | 0.001371 | 0.310758 | 0.95 |
| rs6679677 | Walking pace | A | C | -0.00495 | 0.002098 | 0.100752 | 0.018 |
| rs6739788 | Walking pace | T | A | -0.00323 | 0.002803 | 0.053812 | 0.25 |
| rs6798068 | Walking pace | A | G | -0.00132 | 0.001352 | 0.322011 | 0.33 |
| rs6833591 | Walking pace | G | A | 0.000824 | 0.001332 | 0.347061 | 0.54 |
| rs6992869 | Walking pace | C | T | -0.00186 | 0.001309 | 0.375327 | 0.16 |
| rs7005834 | Walking pace | T | C | -0.00173 | 0.001368 | 0.307358 | 0.21 |
| rs7090530 | Walking pace | A | C | -0.0015 | 0.001292 | 0.602208 | 0.25 |
| rs71508903 | Walking pace | T | C | 0.001416 | 0.001616 | 0.194116 | 0.38 |
| rs731151 | Walking pace | A | G | -0.00379 | 0.001703 | 0.165293 | 0.026 |
| rs7441808 | Walking pace | G | A | 0.00157 | 0.001379 | 0.30079 | 0.26 |
| rs7582694 | Walking pace | G | C | 0.001423 | 0.001514 | 0.773916 | 0.35 |
| rs7583027 | Walking pace | C | A | 0.001554 | 0.00132 | 0.64113 | 0.24 |
| rs7596240 | Walking pace | G | A | 0.00149 | 0.001424 | 0.270128 | 0.3 |
| rs761357 | Walking pace | T | A | -0.00653 | 0.001311 | 0.375175 | 6.40E-07 |
| rs7649344 | Walking pace | C | T | 0.002197 | 0.001272 | 0.456993 | 0.084 |
| rs76518703 | Walking pace | G | A | -0.00658 | 0.002979 | 0.052161 | 0.027 |
| rs76930710 | Walking pace | C | T | 0.002765 | 0.00328 | 0.039381 | 0.4 |
| rs7705526 | Walking pace | A | C | 0.000578 | 0.001367 | 0.326537 | 0.67 |
| rs772920 | Walking pace | G | C | 0.002255 | 0.00134 | 0.335502 | 0.092001 |
| rs7768019 | Walking pace | G | C | -0.00026 | 0.001471 | 0.244003 | 0.86 |
| rs7850258 | Walking pace | G | A | -0.00186 | 0.001344 | 0.668035 | 0.17 |
| rs7936397 | Walking pace | A | G | -0.00055 | 0.001423 | 0.272634 | 0.7 |
| rs79490353 | Walking pace | C | T | -0.01274 | 0.004031 | 0.025264 | 0.0016 |
| rs8054578 | Walking pace | G | A | 0.00178 | 0.001516 | 0.774457 | 0.24 |
| rs8093850 | Walking pace | G | A | 0.001356 | 0.001365 | 0.31764 | 0.32 |
| rs897586 | Walking pace | A | G | 0.002391 | 0.001323 | 0.356899 | 0.071 |
| rs9272426 | Walking pace | G | A | -0.00396 | 0.001292 | 0.452233 | 0.0022 |
| rs9277569 | Walking pace | T | C | -0.00382 | 0.002027 | 0.109462 | 0.059 |
| rs9291444 | Walking pace | T | C | -0.00135 | 0.001269 | 0.470539 | 0.29 |
| rs9347170 | Walking pace | T | C | 0.000841 | 0.001338 | 0.336542 | 0.53 |
| rs9497965 | Walking pace | T | C | 0.001429 | 0.001291 | 0.40946 | 0.27 |
| rs9511151 | Walking pace | A | G | 0.00017 | 0.001336 | 0.345885 | 0.9 |
| rs9557168 | Walking pace | A | G | 0.001192 | 0.001585 | 0.201908 | 0.450001 |
| rs9697210 | Walking pace | A | G | -0.00142 | 0.001797 | 0.145899 | 0.43 |
| rs970987 | Walking pace | A | C | -0.00022 | 0.001343 | 0.662935 | 0.87 |

**Supplementary Table 3.** Genome-wide significant SNPs for Subclinical hyperthyroidism

| SNP | Outcome | EA | OA | BETA | SE | EAF | P |
| --- | --- | --- | --- | --- | --- | --- | --- |
| rs17477923 | Low hand grip strength | T | C | 0.0087 | 0.0085 | 0.7473 | 0.3048 |
| rs2046045 | Low hand grip strength | T | G | -0.0013 | 0.0075 | 0.6044 | 0.8584 |
| rs2983514 | Low hand grip strength | A | G | -0.0068 | 0.0078 | 0.6742 | 0.3841 |
| rs66760320 | Low hand grip strength | T | C | 0.0017 | 0.0085 | 0.2507 | 0.8371 |
| rs8077245 | Low hand grip strength | T | G | 0.0087 | 0.0074 | 0.5262 | 0.2382 |
| rs925488 | Low hand grip strength | A | G | -0.0192 | 0.0078 | 0.669 | 0.01399 |
| rs17477923 | ALM | T | C | 9.00E-04 | 0.0022 | 0.7469 | 0.667 |
| rs2046045 | ALM | T | G | -0.0001 | 0.0019 | 0.6075 | 0.959 |
| rs66760320 | ALM | T | C | 0.0022 | 0.0022 | 0.2497 | 0.3153 |
| rs8077245 | ALM | T | G | -0.0007 | 0.0019 | 0.521 | 0.716 |
| rs925488 | ALM | A | G | -0.0074 | 0.002 | 0.668 | 0.000227 |
| rs17477923 | Walking pace | T | C | 0.000552 | 0.001464 | 0.746814 | 0.709999 |
| rs2046045 | Walking pace | T | G | -0.00158 | 0.001295 | 0.60742 | 0.22 |
| rs2983514 | Walking pace | A | G | 0.000494 | 0.001355 | 0.675441 | 0.719999 |
| rs66760320 | Walking pace | T | C | 0.000547 | 0.001461 | 0.249917 | 0.709999 |
| rs8077245 | Walking pace | T | G | -0.00224 | 0.001267 | 0.52158 | 0.077 |
| rs925488 | Walking pace | A | G | -0.00188 | 0.001344 | 0.667953 | 0.16 |

**Supplementary Table4.** Genome-wide significant SNPs for subclinical hypothyroidism

| SNP | Outcome | EA | OA | BETA | SE | EAF | P |
| --- | --- | --- | --- | --- | --- | --- | --- |
| rs11675342 | Low hand grip strength | T | C | -0.0156 | 0.0074 | 0.4249 | 0.03576 |
| rs12449792 | Low hand grip strength | T | C | -0.0021 | 0.0075 | 0.4638 | 0.780301 |
| rs1382879 | Low hand grip strength | T | C | -0.0005 | 0.0075 | 0.6067 | 0.9473 |
| rs2983514 | Low hand grip strength | A | G | -0.0068 | 0.0078 | 0.6742 | 0.3841 |
| rs597808 | Low hand grip strength | A | G | 0.0099 | 0.0074 | 0.4855 | 0.1771 |
| rs7032019 | Low hand grip strength | A | G | -0.0227 | 0.0085 | 0.6679 | 0.007845 |
| rs75491569 | Low hand grip strength | T | C | 0.0117 | 0.0101 | 0.157 | 0.2477 |
| rs78495697 | Low hand grip strength | T | C | -0.0039 | 0.0129 | 0.089 | 0.764401 |
| rs11675342 | ALM | T | C | 8.00E-04 | 0.0019 | 0.4233 | 0.6611 |
| rs12449792 | ALM | T | C | 0.0105 | 0.0019 | 0.4637 | 4.69E-08 |
| rs1382879 | ALM | T | C | 2.00E-04 | 0.0019 | 0.6099 | 0.9133 |
| rs597808 | ALM | A | G | -0.0179 | 0.0019 | 0.4843 | 2.84E-21 |
| rs7032019 | ALM | A | G | -0.0074 | 0.002 | 0.6673 | 0.000217 |
| rs75491569 | ALM | T | C | -0.003 | 0.0026 | 0.1561 | 0.2439 |
| rs78495697 | ALM | T | C | 0.0016 | 0.0033 | 0.0887 | 0.6185 |
| rs11675342 | Walking pace | T | C | 0.000294 | 0.001282 | 0.423556 | 0.82 |
| rs12449792 | Walking pace | T | C | 0.003761 | 0.001285 | 0.463466 | 0.0034 |
| rs1382879 | Walking pace | T | C | -0.00157 | 0.001297 | 0.609759 | 0.23 |
| rs2983514 | Walking pace | A | G | 0.000494 | 0.001355 | 0.675441 | 0.719999 |
| rs597808 | Walking pace | A | G | -0.00222 | 0.001269 | 0.48408 | 0.081001 |
| rs7032019 | Walking pace | A | G | -0.00188 | 0.001345 | 0.667928 | 0.16 |
| rs75491569 | Walking pace | T | C | -0.00326 | 0.001745 | 0.156211 | 0.061 |
| rs78495697 | Walking pace | T | C | 0.002182 | 0.002226 | 0.088605 | 0.33 |
